# Correlation of percentage changes in platelet counts with recurrence rate following radical nephrectomy

**DOI:** 10.4103/0970-1591.65383

**Published:** 2010

**Authors:** A. Patel, R. Bhavan, B. Somani, G. Nabi

**Affiliations:** Department of Urology, Ninwells University Hospital, Scotland, Dundee, UK

**Keywords:** Platelets, radical nephrectomy, renal cancer

## Abstract

**Objectives:**

To categorize and correlate percentage changes in platelets counts - an objective approach with recurrence rate following radical nephrectomy.

**Materials and Methods:**

All consecutive patients who had radical nephrectomy for localized renal tumor in the period from January 1997 to December 2005 have been included in this study. The data was collected retrospectively. The primary outcome of this study was over all and cancer- specific survival and its correlation with percentage change in platelet count from pre-surgical level. Change in platelets counts was categorized as less than or more than 0-10%, 10-20% and more than 20% from base line (pre-surgery). This was correlated with the follow-up recurrence and disease free survival. Survival distribution were estimated using Kaplan-Meier method, univariate and multivariate regression analyses were performed using Cox proportional hazards models to address the impact of different prognostic factors on survival.

**Results:**

Of the 237 patients treated with radical nephrectomy, pT1, pT2, pT3, and pT4 accounted for 116 (49%), 44 (18.5%), 68(28.7%), and nine (3.8%) cases respectively. The mean tumor size was 6.3 cm (Range: 4-17 cm; SD: 3). The pre-operative platelet count ranged from 82 to 1573 (Mean: 327.5; SD: 171.7). The overall follow-up time ranged from 1-102 months (Mean: 39 months; SD: 27months). There was significant correlation between the recurrence rate and increase in platelets count of more than 20% following radical nephrectomy (*P* value- 0.0001).

**Conclusions:**

Categorization of platelets changes following radical nephrectomy for localized renal cell carcinoma, in particular, a change in more than 20% can accurately predict recurrence and cancer specific survival following radical nephrectomy for localized renal cell carcinoma.

## INTRODUCTION

The incidence of renal cell carcinoma is increasing with approximately 208,000 new cases and 102,000 deaths resulting from this disease throughout the world.[1] Even with the introduction of promising new treatments, surgery remains the only therapy that is curative for the localized disease;[1–3] 30 to 50% of patients relapse following initial curative-intended radical surgery,[2] moreover, metastatic disease is present in 30% of the patients at the time of initial presentation. Thus, metastatic renal cell carcinoma remains a major challenge for the uroncologists. Although a minority is being cured with immunotherapy, majority of the patients with metastatic disease will die of their disease with no benefit from the available modalities of treatment at present. The main focus of the current research, in addition to the development of novel therapeutic agents is to identify prognostic factors that can help physicians select patients who will or not benefit from the adjuvant treatment following the local surgical treatment.

The utility of prognostic markers research is highly desirable in predicting risk of renal cell carcinoma, response to therapy and identification of molecular pathways that may serve as targets of novel therapeutic agents. Based on the current status of knowledge, prognostic markers can be: tumor related factors, clinical symptoms, laboratory findings, and pathological factors, in particular the Fuhrman grade of renal cell carcinoma.[4]

Thrombocytosis has been implicated in several malignancies.[5] In localized and metastatic renal cell carcinoma, thrombocytosis is considered a negative prognostic factor.[1,3,6,7–9] The mechanism of thrombocytosis in renal cell carcinoma is poorly understood.[1] Thrombocytosis may reflect a cascade of biological events that are related to tumor aggressiveness.[10] Platelets are involved in the angiogenesis process that is important in the pathogenesis of renal cell carcinoma.[1] Platelets may provide adherence for endothelial wall.[1,6] They are also a source of vascular endothelial growth factor that plays an important role in the process of many diseases, as well as tumor angiogenesis.[11] Patients with malignancy have been noted to have high levels of intracellular vascular endothelial growth factor.[11] Thrombospondin, a platelet secreted protein, has also been implicated in metastatic tumor spread, due to its aggregating function in essential thrombocythaemia.[12] In renal cell carcinoma the release of cytokines can stimulate the production of megakaryocytes in the bone marrow and thereby elevate the platelet count and indirect secretion of vascular endothelial growth factor.[11,13] In particular, interleukin-6[14] with its proinflammatory abilities is able to stimulate hematopoiesis and proliferation of megakaryocyte progenitors.[13]

While previous studies have reported poor survival in patients with preoperative or postoperative thrombocystosis, the definition of thrombocytosis varied between the different reports.[1,6] This makes generalizability of the findings difficult to prognosticate the disease. In the present study, we evaluate the significance of percentage change in platelet counts from pre-surgical patients who underwent a radical nephrectomy for localized renal cell carcinoma. We compare our findings with other prognostic variables, and correlated the percentage change in platelet count with follow-up recurrence and disease free survival.

## MATERIALS AND METHODS

### Patients population

A total of 237 consecutive single-institution patients who underwent radical nephrectomy for localized renal tumor between January 1997 and December 2005 were retrospectively included in this study. Records of all the patients were retrieved from the hospital information system. In all the cases TNM stage, Fuhrman grade, tumor size, nodal invasion, histological subtype and platelet count before and at follow-up visits were recorded. A record of disease status at last follow-up was obtained in all the patients. Follow-up following radical nephrectomy consisted of one postoperative visit at six to eight weeks and then performed every six months for a minimum of two years. Subsequently, minimum follow-up consisted of annual visits. At each visit, patients were assessed clinically and had blood chemistry, chest x-ray with CT scan or abdominal ultrasound. The cause of death was either obtained from the medical chart or was obtained from the death certificate in a retrospective fashion from Registrar General Office in Scotland. RCC-specific mortality included deaths that were directly attributable to kidney cancer.

### Primary outcome and statistical analysis

The primary outcome of this study was over all and cancer- specific survival and its correlation with percentage change in platelet count from pre-surgical level. We choose an arbitrary 10% change and platelets counts were categorized as less than or more than 0-10%, 10-20% and more than 20% from the base line (pre-surgery). This was correlated with the follow-up recurrence and disease free survival. An arbitrarily cut-off level of 10% change was chosen based on two assumptions: the first, categorization using a figure of less than 10% was likely to increase the number of comparison groups leaving small number of patients in each group. The second, we expected that the event rates used for measuring outcomes such as recurrence rate based on the existent literature (30% following curative surgery for localized renal cell carcinoma) would be extremely small, if categorization was done using an arbitrarily level of less than 10%. Survival distribution were estimated using Kaplan-Meier method, univariate and multivariate regression analyses were performed using Cox proportional hazards models to address the impact of different prognostic factors on survival.

## RESULTS

Patient characteristics are shown in the Table 1. The age ranged from 17 to 88 years (mean: 64). Of the 237 patients treated with radical nephrectomy, pT1, pT2, pT3, and pT4 accounted for 116 (49%), 44 (18.5%), 68 (28.7%), and 9 (3.8%) cases respectively. The mean tumor size was 6.3 cm (Range: 0-17 cm; SD: 3). Clear-cell histology was present in 206 (87%) patients, other histologies accounted for 31 (13%) patients. Fuhrman II (131 patients, 55%) and III (54 patients, 23%) represented the most frequent tumor grades. Node-positive disease was diagnosed in four cases.

**Table 1:** Baseline characteristics of patients treated with radical nephrectomy for localised renal cell carcinoma

Variable					
Total cohort			237		
Age (Mean and standard deviation)			64± 20		
Sex Stage					
T1			116		
T2			44		
T3			68		
T4			9		
Grade					
I			43		
II			131		
III			54		
IV			9		
Platelet count (Mean and SD)			327.5 ± 171.7		
Follow up (months) Mean and SD			41 ± 25		
No recurrences			149		

Patients alive with recurrences and change in percentage of platelets count from the baseline					
>20%	>10-20%	>0-10%	<0-10%	<10-20%	<20%
36	2	4	3	1	5

Patients who died with or without recurrences and change in percentage of platelets count from the baseline					
Deaths due to unrelated causes			Deaths due to renal cell carcinoma		
		
11			29			
		Increase in platelets percentage	No change in Platelets percentage	Unknown	
		23	1	5	

### Patients with no recurrences on follow-up [Figure 1]

All the patients had preoperative platelets count determination and it ranged from 82 to 1573 (Mean: 327.5; SD: 171.7). There were no recurrences seen in 149 patients at a follow-up ranging from 6-108 months (Mean: 41 months; SD: 25 months). Three patients in this group died due to unrelated reasons. There were three and five patients in this group with increase in platelets count of more than 20% and 10% respectively. Majority of the remaining patients in this group (97/138; 70%) had a decrease in platelets counts by more than 10%.

**Figure 1:**
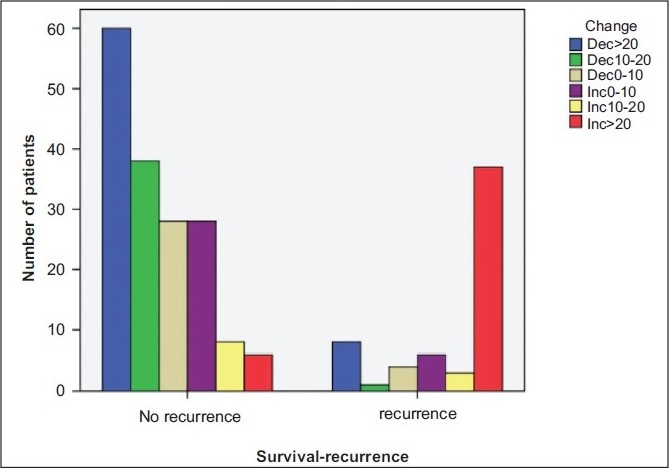
Platelets counts change and recurrences following radical nephrectomy

### Patients with recurrences and death on follow-up

Of the remaining 88 patients, 29 (32%) died due to metastatic diseases and eight (9%) died due to unrelated causes. For those who died of RCC, the mean and median times to RCC-specific death were respectively 20.6 months and 12 months respectively. Of the 29 patients who died due to metastatic disease, 17 (58.6%) had a platelets percentage change of more than 20% as measured on follow-up. Only one patient in this group did not show rise in percentage platelets count prior to recurrence and death. Six patients had a percentage platelets counts rise between 0-10% at different stages of follow-up prior to death. In five patients change in platelets count could not be calculated due to insufficient data.

### Patients with recurrences and surviving on follow-up

Fifty one patients were alive with recurrences [Figure 1]. Of these, 36 had percentage platelets counts rise by more than 20%. Two and four patients had a rise in platelets count of 10-20% and 0-10% respectively. Nine patients had a decrease in the percentage change in platelets count ranging from 0-20%. In 42 patients who are alive with recurrences [Table 1], at least one value of increase in platelets count of more than 10% preceded the recurrence. Table 2 shows distribution of stage and grade of the disease in this group of patients with increase in platelets percentage and recurrences.

**Table 2:** Grade and stages of tumours in the category of patients with change (increase) in platelets counts percentage and recurrence on follow-up

Grade	Stage of tumour						
	
	1A	1B	2	3A	3B	4	Total
1	0	0	2	0	0	0	2
2	1	5	5	3	5	0	19
3	1	0	6	4	5	2	18
4	0	0	0	1	1	1	3
Total	2	5	13	8	11	3	42

### Statistical analysis

Survival estimates according to the previously described categories of percentage change in the platelets counts is shown in Table 1 and Table 2. Kaplan-Meier method, univariate and multivariate regression analyses were performed using Cox proportional hazards models to address the impact of different prognostic factors on survival. A change in platelets of more than 20% was found to be a significant prognostic factor with P Value of 0.001 (SE-0.87). Grade of the tumor, stage of the disease and platelets count of more than 10-20% were found to be less significant with p-values of 0.473 (SE-0.138); 0.01 (SE-0.292) and 0.184 (SE-0.747) respectively. Similarly, age and sex were found not to be statistically significant.

## DISCUSSION

### Principal findings of study

The pertinent observation from the present study highlights a strong correlation between percentage change in the platelets counts and follow-up recurrences after radical nephrectomy for localized renal cell carcinoma. In majority of the patients with recurrences, platelet counts showed a consistent change from pre-surgical level and at least one change of more than 0-10% preceded the radiologically obvious recurrences. This adds to already an already established concept of poor prognostic value of thrombocystosis in the outcome of renal cell carcinoma. In this study we suggest an empirical scheme for categorization of platelets changes in patients with renal cell carcinoma, which if reproduced by subsequent studies can bring objectivity to this key prognostic factor. The design of the present study would help in clarifying, communicating and translating changes in platelets counts following radical nephrectomy into implementation research and prognostic models.

### Existent literature on the thrombocytosis and prognosis of renal cell carcinoma

Several studies have shown thrombocytosis to be associated with a poor prognosis in localized and metastatic renal cell carcinoma.[1,3,6,15] Symbas *et al*, in their study noted thrombocytosis to be associated with a poor outcome and shorter life expectancy in patients with metastatic renal cell carcinoma.[3] They concluded that even when tumor stage, grade and cell type were controlled, thrombocytosis was an important prognostic indicator.[3] Their data showed that patients with metastatic renal cell carcinoma, who received adjuvant therapy and had a persistently normal platelet count, had a 64% increase in life expectancy in comparison to those patients with thrombocytosis.[3]

O’Keefe *et al*, also concluded thrombocytosis as an independent prognostic factor in their study.[6] They looked into whether thrombocytosis was predictive of death from renal cell carcinoma after radical nephrectomy was performed for early stage disease, with the intention to cure.[6] They reviewed the records of 204 patients who had undergone a radical nephrectomy during June 1993-2000.[6] Thrombocytosis was classified as a platelet count greater than 40,000/mm^3^. They reported thrombocytosis as being associated with reduced survival in patients with renal cell carcinoma.[6] In 178 patients without thrombocytosis the overall and cause-specific death rates were reported as 15.2% and 7.3% respectively. In the 26 patients with thrombocytosis the overall and cause specific death rates were noted to be 50% and 42% respectively. There was a 5.75-fold increase in cancer specific death rate in those with thrombocytosis compared to those with normal platelet counts.[6] Bensalah *et al*,[1] in their study found that patients with a platelet count >45,000/mm^3^ were approximately twice as likely to die of renal cell carcinoma. They noted thrombocytosis to be independent of stage, grade and performance on multivariate analysis.[1] Platelet count correlated with tumor aggressiveness, and thrombocytosis was noted to have an adverse effect on survival, in both local and advanced renal cell cancer.[1] Gogus *et al*,[15] in their study of 151 patients evaluated the significance of thrombocytosis in determining survival in patients with localized renal cell carcinoma who had undergone radical nephrectomy.[15] Preoperative thrombocytosis was highlighted to be a significant predictor in determining prognosis in patients with localized renal cell carcinoma.[15]

All these studies reported in the literature highlight and validate the use of thrombocytosis as a prognostic indicator in renal cell carcinoma, however, what level of platelets count should be used to prognosticate the disease, lacks consensus. In the present study, a categorization approach of platelets changes showed that all patients with increase in platelets count by 20% from the baseline had recurrence of renal cell carcinoma.

In a complex disease with unpredictable natural history such as renal cell carcinoma, prognostic models such as developed by Memorial Sloan-Kettering Cancer centre serve as an important source for patients’ risk assessment and counseling.[19] With the introduction of Vascular Endothelial Growth Factor (VEGF)-targeted therapy, the focus of prognostic modeling has broadened to include molecular markers including alteration in the von Hippel-Lindau gene status, in particular, loss of gene function.[16] Moreover, expression of these molecular targets may help in predicting response to targeted therapy in a subset of patients population.[17] These models need external validation prior to their application in the clinical practice, however. In a large prospective study, a subset analysis of patients receiving VEGF- targeted therapy for metastatic renal cell carcinoma, a baseline platelets counts of > 300K/µl was reported to be a poor prognostic factor for progression free survival,[18] further studies are needed to confirm the role of systemic treatment. Till then these patients would better served by close follow-up following radical nephrectomy, with curative intention.

### Limitations and weaknesses of the study

The main limitation of the present study is relatively short follow-up. The study cohort selected in a retrospective manner may not represent a homogenous population and hence results may be biased and make it difficult to achieve an external validity. Nevertheless, we believe that our study raises an important observation which needs further prospective evaluation in a multicenter setting.

## CONCLUSION

Categorization of platelets changes following radical nephrectomy for localized renal cell carcinoma is an objective method of measuring thrombocytosis. These changes, in particular - an increase of more than 20% can accurately predict recurrence and cancer-specific survival

## References

[1] Bensalah K, Leray E, Fergelot P, Rioux-Leclercq N, Tostain J, Guillé F (2006). Prognostic value of thrombocytosis in renal cell carcinoma. J Urol.

[2] Ljungberg B (2007). Prognostic markers in renal cell carcinoma. Curr Opin Urol.

[3] Symbas NP, Townsend MF, El-Galley R, Keane TE, Graham SD, Petros JA (2000). Poor prognosis associated with thrombocytosis in patients with renal cell carcinoma. BJU Int.

[4] Fuhrman SA, Lasky LC, Limas C (1982). Prognostic significance of morphologic parameters in renal cell carcinoma. Am J Surg Pathol.

[5] Levin J, conley CL (1964). Thrombocytosis associated with malignant disease. Arch Intern Med.

[6] O’Keefe SC, Marshall FF, Issa MM, Harmon MP, Petros JA (2002). Thrombocytosis is associated with a significant increase in the cancer specific death rate after radical nephrectomy. J Urol.

[7] Verheul HM, Pinedo HM (2003). The importance of platelet counts and their contents in cancer. Clin Cancer Res.

[8] Suppiah R, Shaheen PE, Elson P, Misbah SA, Wood L, Motzer RJ (2006). Thrombocytosis as a prognostic factor for survival in patients with metastatic renal cell carcinoma. Cancer.

[9] Ito K, Asano T, Yoshii H, Satoh A, Sumitomo M, Hayakawa M (2006). Impact of thrombocytosis and C-reactive protein elevation on the prognosis for patients with renal cell carcinoma. Int J Urol.

[10] Daly ME, Makris A, Reed M, Lewis CE (2003). Hemostatic regulators of tumor angiogenesis: a source of antiangiogenic agents for cancer treatment?. J Natl Cancer Inst.

[11] Jacobsen J, Grankvist K, Rasmuson T, Ljungberg B (2002). Prognostic importance of serum vascular endothelial growth factor in relation to platelet and leukocyte counts in human renal cell carcinoma. Eur J Cancer Prev.

[12] Tuszynski GP, Gasic TB, Rothman VL, Knudsen KA, Gasic GJ (1987). Thrombospondin, a potentiator of tumor cell metastasis. Cancer Res.

[13] Schuler M, Peschel C, Schneller F, Fichtner J, Färber L, Huber C (1996). Immunomodulatory and hematopoietic effects of recombinant human interleukin-6 in patients with advanced renal cell cancer. J Interferon Cytokine Res.

[14] Hollen CW, Henthorn J, Koziol JA, Burstein SA (1991). Elevated serum interleukin-6 levels in patients with reactive thrombocytosis. Br J Haematol.

[15] Göğüş C, Baltaci S, Filiz E, Elhan A, Bedük Y (2004). Significance of thrombocytosis for determining prognosis in patients with localized renal cell carcinoma. Urology.

[16] Golshayan AR, Brick AJ, Choueiri TK (2008). Predicting outcome to VEGF-targeted therapy in metastatic clear-cell renal cell carcinoma: Data from recent studies. Future Oncol.

[17] Lam S, Leppert JT, Yu H (2005). Expression of the vascular endothelial growth factor family in tumor dissemination and disease free survival in clear cell renal cell carcinoma. J Clin Oncol.

[18] Golshayan A, Chouriei TK, Elson P (2007). Clinical factors associated with outcome in metastatic renal cell carcinoma patients treated with VEGF-targeted therapy. J Clin Oncol.

[19] Lane BR, Kattan MW (2005). Predicting outcomes in renal cell carcinoma. Curr Opin Urol.

